# The effect of survey method on survey participation: Analysis of data from the Health Survey for England 2006 and the Boost Survey for London

**DOI:** 10.1186/1471-2288-10-83

**Published:** 2010-09-27

**Authors:** Jennifer S Mindell, Sarah Tipping, Kevin Pickering, Steven Hope, Marilyn A Roth, Bob Erens

**Affiliations:** 1 Department of Epidemiology & Public Health, UCL (University College London), 1-19 Torrington Place, London WC1E 6BT, UK; 2 National Centre for Social Research (NatCen), 35 Northampton Square, London EC1V 0AX, UK

## Abstract

**Background:**

There is a need for local level health data for local government and health bodies, for health surveillance and planning and monitoring of policies and interventions. The Health Survey for England (HSE) is a nationally-representative survey of the English population living in private households, but sub-national analyses can be performed only at a regional level because of sample size. A boost of the HSE was commissioned to address the need for local level data in London but a different mode of data collection was used to maximise participant numbers for a given cost. This study examines the effects on survey and item response of the different survey modes.

**Methods:**

Household and individual level data are collected in HSE primarily through interviews plus individual measures through a nurse visit. For the London Boost, brief household level data were collected through interviews and individual level data through a longer self-completion questionnaire left by the interviewer and collected later. Sampling and recruitment methods were identical, and both surveys were conducted by the same organisation. There was no nurse visit in the London Boost. Data were analysed to assess the effects of differential response rates, item non-response, and characteristics of respondents.

**Results:**

Household response rates were higher in the 'Boost' (61%) than 'Core' (HSE participants in London) sample (58%), but the individual response rate was considerably higher in the Core (85%) than Boost (65%). There were few differences in participant characteristics between the Core and Boost samples, with the exception of ethnicity and educational qualifications. Item non-response was similar for both samples, except for educational level. Differences in ethnicity were corrected with non-response weights, but differences in educational qualifications persisted after non-response weights were applied. When item non-response was added to those reporting no qualification, participants' educational levels were similar in the two samples.

**Conclusion:**

Although household response rates were similar, individual response rates were lower using the London Boost method. This may be due to features of London that are particularly associated with lower response rates for the self-completion element of the Boost method, such as the multi-lingual population. Nevertheless, statistical adjustments can overcome most of the demographic differences for analysis. Care must be taken when designing self-completion questionnaires to minimise item non-response.

## Background

Robust local level data on health and health behaviours is needed to assess local area health outcomes, to develop and monitor policies and interventions aimed at determinants of health and to plan local health and other services. For example, data on smoking prevalence overall and by population sub-group are needed to assess tobacco control policies and smoking cessation support and target where these should best be focused. Alternatives, such as synthetic estimates, are not always appropriate as levels of precision are often low and they cannot be used to monitor changes in response to local interventions [1].

The Health Survey for England (HSE) is an annual health examination survey of a new, nationally-representative sample of the general population each year. It provides reliable data on a broad range of health topics [2]. The HSE was not designed to provide local area data: the sample sizes (there were 16,000 adults and 7,300 children in 2006) are too small for reliable estimates below the regional level. The HSE sample in a Primary Care Trust (PCT) would need to be supplemented to perform PCT-level analyses. The optimum design for the boost sample would be one that matched the main HSE sample exactly, however, this would also have been more expensive. Using a self-completion questionnaire for most of the data collection maximises the sample size for a given budget, whilst retaining the original sample design. This approach is used for the Welsh Health Survey [3] (WHS), being less expensive than face-to-face interviews. A pan-London consortium, led by the London Health Observatory, commissioned a boost to the HSE in London to obtain survey results for London as a whole and for each of its 31 PCTs [4].

Using interviews and self-completion questionnaires for health surveys in the same geographical area provided an opportunity to examine differential response rates, and how this might impact results. A decreasing trend in survey participation rates has been reported in the USA [5] and several European countries over the last decades [6-11]. A 2007 review attributes some of this decrease to increasing numbers of research studies plus the proliferation of political polls and marketing by telephone calls and surveys that may look similar to scientific surveys [12]. Survey information arriving by post or by telephone may be considered "junk" together with unsolicited mail or calls from commercial sources. Low response rates are important since respondent characteristics may differ from those of non-respondents, which can introduce bias into survey estimates [13]. In addition, response rates have been found to vary between modes [14], which could add substantial differential response bias to results combined across modes. However, overall response may mask variation in response within a survey.

Item non-response relates to particular questions which are missed, even though the respondent has taken part in the survey. It can be an indication of a range of issues, such as the design, topic and placement of the question, the characteristics of the participant and the mode of data collection [15]. Understanding the relationship between mode and level of non-response when surveying health and health behaviours is vital if mixed mode data collection is to be used in the future. This paper examines differences in response rates and characteristics of participants and item non-response. A companion paper investigates the effects of mode and context of the survey method on question responses (manuscript submitted).

## Methods

### Participants

The Health Survey for England (HSE) is an annual cross-sectional survey of a nationally representative sample of the English population living in private households. Participants are visited by interviewers, who measure height and weight and collect household and individual level data using computer-assisted personal interviews (CAPI). Participants who agree are visited on a separate occasion by nurses, who obtain further measurements and biological samples. The interviewer's visit includes a short self-completion booklet, which includes questions on perceived social support and the General Health Questionnaire (GHQ12, a measure of psychological health) questions [16].

The method used for the London Boost was adapted from the WHS method. It involved leaving self-completion questionnaires rather than face-to-face CAPI. Interviewers visited selected addresses to carry out the household interview (using a paper questionnaire), and recruit household members. Interviewers returned to collect completed individual questionnaires and encourage non-responding household members to participate, calling at the address a few times if necessary. The household interview included questions on household members' sex, age, household type, activity of the Household Reference Person (HRP, the person in the household responsible for the accommodation, or if more than one person, the person with the highest income), occupation of the HRP and whether any household members smoked. The interviewer measured the height and weight of all consenting participants who were present at either visit. There was no nurse visit.

To encourage participation, the self-completion questionnaire was kept reasonably short: only a sub-set of topics and questions from the HSE were included.

The sample was selected from the small-user Postcode Address File (PAF), which is a list of all the mail delivery addresses in England. The PAF has very good coverage of private addresses, excluding fewer than 1% of households. To increase the precision of the sample, prior to selection, the PAF was sorted by local authority (PCT within London) and the percentage of households with a head of household in a non-manual occupation (Socio-Economic Groups 1-6, 13), taken from the 2001 Census.

The samples for both the HSE and London Boost were drawn using a two-stage, stratified sampling procedure. Primary Sampling Units (PSU) were single or grouped postcode sectors: postcode sectors with fewer than 500 addresses were combined with neighbouring sectors before selection to avoid clustering of sampled addresses. For the national HSE 2006 Core sample, a sample of 720 PSUs was drawn at the first stage with probability proportional to the total number of addresses within them; 102 PSUs fell within London. At the second stage, a fixed number of addresses was selected per PSU. This design gives each address an equal chance of being selected, making the sample of addresses representative of all addresses in England.

For the London Boost sample, 202 PSUs were selected at the first stage with unequal probability to ensure 6 PSUs were selected per London PCT (more in boroughs that had commissioned larger boosts). At the second stage, a fixed number of addresses were selected per PSU, with more addresses selected in inner London, where response rates were expected to be lower. The unequal selection probabilities meant address selection weights were required for analysis to make the sample representative of London.

People living in institutions were not included in either survey. All adults (aged 16 and over) up to ten and two children (aged 0-15 years) in each household were eligible to participate. Where there were more household members than these, participants were selected at random using a Kish grid. The household protocol was the same across the Boost and the Core except that the household questionnaire in the Boost was shorter and completed by the interviewer using pen and paper, rather than CAPI. Interviewers are required to make at least four attempts at contact, at different times of day and day of the week, before a household is considered a non-responder. The mean number of calls made to non-responding addresses in 2006 was 7.6.

All London Boost Survey participants ('Boost') and all Core HSE participants resident in London ('Core') aged 16+ were included in the analyses. More detail is available elsewhere [17]. Ethical approval was obtained for both surveys from the London Multi-centre Research Ethics Committee.

### Statistical Analysis

#### Non-response

Comparison was made between the overall response levels of households and individuals to the Boost and Core surveys, to assess whether differences in mode affected response. The impact of differential response rates on the sample composition was assessed by comparisons of respondent characteristics.

#### Characteristics of respondents

The effects of differential response on sample composition were investigated further by comparing the socio-demographic characteristics of the two samples. Non-response weights were not applied during this analysis, as the aim was to identify differences in the achieved samples. However, selection weights were required to make the two samples comparable, as the sample design of the London Boost meant it was not representative of the London population. Area-level variables included: PCT Spearhead status (Spearhead PCTs contain the 20% most health-deprived local authorities and receive extra funding to tackle health inequalities) [18]; PCT location in inner or outer London; quintile of index of multiple deprivation (IMD 2004) [19]; the proportion of persons in the PSU belonging to a non-white minority ethnic group; and the proportion of household heads in the PSU from non-manual occupations (both based on data from the 2001 Census).

Further comparisons were carried out to examine whether differences between the Core and Boost samples remained once non-response weights had been applied. These weights correct for both unequal selection probabilities and the effects of differential non-response. The two samples were weighted separately using the same weighting procedure, which corrected for differential response by age, sex, household type and inner/outer London, and is described further in the Appendix and elsewhere [17].

#### Item non-response

The number of missing data items (item non-response) by mode was examined by comparing the number of survey participants who had not answered (i.e. refused or skipped) individual questions.

Since both samples were clustered, stratified and weighted, the analysis was run in Stata 10 using the 'svy' command to account for the complex sample design. Data from men and women were analysed separately to reduce clustering within households.

## Results

### Household response rates

963 households were interviewed in the Core London sample and 3,882 households in the Boost. There were small but significant differences in response rates between the two modes for households (58% Core; 61% Boost; p < 0.001, Table 1). The national response rate for the 2006 HSE was 68% [13]. The Boost had a significantly higher response rate in outer London PCTs than the Core. There was much overlap between Spearhead status and location of PCT: 16 of the 18 outer London PCTs were also non-Spearhead PCTs, hence the response rate in non-Spearhead PCTs was also significantly higher for the Boost sample. The Boost method worked better in less deprived areas, while the HSE performed better in slightly more deprived areas (but there was no significant difference in the most deprived areas). There were few differences in response by the proportion of minority ethnic residents.

**Table 1:** Household response rates by sample type and area characteristics

	Household response rates		
	**Core **^**a**^ %	**Boost **^**b**^ %	Significance P-value
*PCT spearhead status*			
Non-Spearhead	55	62	< 0.001
Spearhead	63	61	0.463
*Location*			
Inner London PCTs	60	60	0.687
Outer London PCTs	57	62	0.001
*Index of Multiple Deprivation (IMD) (quintiles)*			
Least deprived (< 11.9)	52	62	0.001
2^nd ^least deprived (11.9 to 19.2)	58	62	0.163
Middle quintile (19.3 to 27.7)	54	62	0.009
2^nd ^most deprived (27.7 to 37.9)	65	59	0.037
Most deprived (> 37.9)	61	62	0.644
*Proportion of minority ethnic population (quintiles)*			
Lowest density (< 12.1%)	56	59	0.253
2^nd ^lowest density (12.1-19.0)	56	62	0.078
Middle quintile (19.1-27.2)	58	63	0.058
2^nd ^highest density (27.2-39.6)	55	61	0.045
Highest density (> 39.6%)	63	61	0.515
*Proportion of non-manual heads of households (quintiles)*			
Lowest density (< 53.0)	65	63	0.632
2^nd ^lowest density - 53.0 to 61.8	60	59	0.870
Middle quintile - 61.9 to 69.7	57	59	0.535
2^nd ^highest density - 69.8 to 78.0	54	64	0.001
Highest density (> 78.0)	53	61	0.007
			
All	58	61	0.005

^a ^unweighted ^b ^weighted for selection

### Response rates of individuals within co-operating households

There were larger differences in the participation rates of eligible individuals within responding households (Table 2). Individuals in the Core were more likely to give a productive interview once the household had responded. There were 1,841 eligible adults living in the 963 Core responding households, of whom 1,569 (85%) gave a productive interview. The 3,882 responding households in the Boost sample contained 7,714 eligible adults, of whom 5,004 (65%) completed a questionnaire. Older people were more likely to respond in each sample. Individuals in multi-adult households were least likely to respond; this effect was greater for the Boost than for the Core.

**Table 2:** Individual response rates within co-operating households

	Individual response rates		
	**Core** **%**	**Boost**^**a**^ **%**	**Significance** **p-value**

**Age group**			

16-34	80	58	< 0.001

35-54	87	70	< 0.001

55+	91	73	< 0.001

**Household composition**			

1 adult 16-59, no children	100	78	< 0.001

2 adults 16-59, no children	84	63	< 0.001

Small family	90	72	< 0.001

Large family	88	64	< 0.001

Large, adult household	72	54	< 0.001

2 adults, 1+ aged 60+, no children	91	74	< 0.001

1 adult aged 60+, no children	98	79	< 0.001

^a ^Boost sample weighted by address selection weight

### Item non-response

The Core survey had lower levels of item non-response than the Boost for both the household and individual questionnaires. Within the Boost survey there were fewer missing items for the household component, where the interviewer carried out the short household interview on paper, than the self-completion component. The Boost had higher levels of item non-response than the Core, although the level of item non-response for the Boost was generally low (< 5%) for the majority of the questions. There was, however, a wide range in the amount of item non-response (Table 3). Levels of item non-response were lower for straightforward questions with simple answer categories, such as general health. They were higher for more sensitive questions, such as ethnicity and economic activity, and for complex modules such as education and physical activity.

**Table 3:** Levels of item non-response for key variables from the individual questionnaire by sample type

	Participants with item missing	
	Core %	Boost %
Participant ethnicity	0.4	2.5
Marital status	0.1	2.1
Participant economic activity	0.3	5.9
Highest education qualification	0.3	9.2
Cigarette smoking status	0.9	2.4
Frequency drunk alcohol in last 12 months	1.1	2.1
Grouped portions of fruit eaten yesterday	0.1	3.0
Self-assessed general health	0.0	1.2
Long-standing illness or disability	0.0	1.9
Limiting long-standing illness or disability	0.0	1.9
Summary physical activity level	0.2	16.1
		
*Base (unweighted)*	*1,569*	*4,942*

The educational qualification question had high item non-response; 9% of the Boost sample did not complete it compared with less than 1% of the Core. The proportion missing for participant economic activity was also high (6% Boost, 0.3% Core).

Some participants followed the filtering incorrectly and skipped a follow-up question they should have answered. 13% of current smokers in the Boost (compared with <1% Core) did not record the number of cigarettes they smoked, and 9% of Boost participants who had drunk alcohol in the last seven days did not give the number of units drunk on the heaviest day, compared with 1% of Core participants.

Higher levels of item non-response in the Boost resulted in more missing data in the 'derived' summary variables used in analysis. These variables combine responses from more than one question: missing data from any one of the component questions produces a missing result for the derived variable. The physical activity derived variable summarises the responses made to a long series of questions; this derived variable was missing for 0.2% Core but 16% of Boost participants.

Although the levels of item non-response were more similar for the social support and GHQ12 questions asked by self-completion in both the Core and Boost surveys, the Boost item non-response (2.9% and 3.5%) was still higher (2.4% and 1.2%).

### Socio-demographic characteristics of participants

After applying selection but not non-response weights, the demographic profiles of the achieved Core and Boost samples were very similar in age and sex (Table 4). Both samples slightly under-represented younger men and over-represented older people compared with the London population. Both had a higher proportion of women than men. This pattern was more evident in the Boost sample, but the difference was not significant.

**Table 4:** Demographic comparison of boost and core samples with the London population

	MEN			WOMEN		
	Core %	Boost %	Population %	Core %	Boost %	Population %
*Sex*	47	44	49	53	55	51
*Age (grouped)*						
16-24	12	12	15	12	13	14
25-34	23	20	25	22	21	24
35-44	22	21	22	24	22	20
45-54	15	18	15	15	16	15
55-64	13	13	11	11	12	11
65+	15	15	13	16	16	16
*Ethnicity*						
White	64	69	72	63	70	72
Mixed	2	2	2	3	3	2
Asian	19	16	13	17	14	12
Black	13	9	9	14	10	10
Chinese/other	3	4	3	4	4	4
						
*Base (unweighted) ^a^*	*736*	*2202*	*3667744*	*833*	*2726*	*3745524*
*Base (weighted) ^b^*	*768*	*2302*		*870*	*2871*	

*^a ^*Bases vary but are of similar sizes; those show are for age group.

^b ^Only selection weights were applied in this analysis.

Further comparisons were carried out on data weighted for non-response. There was a significant difference in the ethnicity of participants, with more non-White participants in the Core sample than in the Boost. The Boost sample, however, was the closer of the two to the ethnic distribution of the London population. The differences in the ethnic profile were no longer significant once non-response weights were applied (Table 4).

There were also significant differences in participants' educational qualifications (Table 5). Boost participants had higher qualifications than Core participants as a percentage of all participants who provided a valid answer, but similar levels as a proportion of all participants (Table 4); these differences persisted even after non-response weights were applied. A far lower proportion of Boost participants reported no qualifications (Core 25%, Boost 15%).

**Table 5:** Socio-economic characteristics of participants by sample type

	Core %	Boost %	Significance p value
*Highest educational qualification*			
Higher degree/Degree/NVQ4/5	35	34	< 0.001
NVQ3/GCE A Level equiv - any grade	11	13	
NVQ1/2 GCE O Level equiv - any grade	25	24	
Other (e.g. City and Guilds, RSA/OCR, BTEC)	4	3	
No qualification	25	15	
Missing	0.3	9	
*Economic activity*			
Full time study	7	8	0.005
In paid work	56	52	
Looking for work	3	3	
Ill (Long and short term)	4	4	
Retired	18	14	
Looking after home	11	10	
Doing something else	1	3	
Missing	0	6	

*Base (unweighted)*	*1,569*	*4,942*	

There were no significant differences in sample profile by participants' current economic activity (Table 5), marital status, household composition, current economic activity of the HRP, NS-SEC of HRP, area-level deprivation indicators, or PCT Spearhead status.

## Discussion

This study provides an opportunity to compare two survey modes regarding response rates, participant characteristics, and item non-response. The household response rates were slightly better for the Boost, whereas the individual response rates were considerably better for the Core. Both were lower than the national HSE 2006 response rate, although response rates tend to be lower in large metropolitan areas like London.

### Response rates

The household response rate was higher for the Boost sample than the Core sample.

The amount of time the interviewer was required to spend in the respondent's home may be a factor in this difference; the household interviews for the Core and Boost samples both ran to around 10 minutes, however, the Core household CAPI interview leads on directly to the individual CAPI interview, which could last up to an hour. Therefore one possible explanation for the differences in household response is that, in comparison with the Core sample, Boost households only had to commit themselves to a short interview.

The reasons the Boost performed more poorly than the Core in terms of individual response rates are unclear. The presence of the interviewer and their ability to motivate household members to participate may partially account for the higher Core individual response rate [15]. The interactive nature of the face-to-face interview may also prove more interesting for participants compared with answering 'exam-type' questions. Moreover, the interviewers used in the Core survey were generally recruited from the more experienced interviewers among the fieldstaff, compared with those who conducted the Boost, since the Boost interview was much shorter and more straightforward. Whilst the London Boost was designed to elicit the highest response possible from individuals within responding households (e.g. by having interviewers return to households to collect completed questionnaires, rather than relying on participants returning them by post), interviewers had less influence over whether individuals completed the self-completion Boost questionnaires compared with the Core face-to-face interviews. Moreover, the Boost survey design allowed for a household interview to be conducted without any individual interviews. While this is also allowed in the Core, it is very unlikely to occur because the person who responds to the household interview usually goes on to complete an individual interview.

For participant characteristics, comparison of the two samples showed few significant differences between the socio-demographic characteristics of the achieved Core and Boost households and individual adults, except that Boost participants appeared to be better educated than Core participants. We might expect this because those with poor reading skills may be put off by the self-completion format. After applying non-response weights, one would expect both samples to provide close correspondence with the London population for the characteristics examined. This was the case, apart from education.

### Item non-response

Item non-response was generally low for both survey modes. High item non-response is an indicator of poor data quality [20]. Item non-response is generally higher for paper self-completion methods than electronic self-completion or interviewer-assisted methods [16,21] the interviewer can encourage the participant to answer and inadvertently missed items are reduced because the electronic CAPI questionnaire automatically filters to the correct next question. This indicates that when self-completion paper questionnaires are to be used successfully, it is vital that the questions and layout are well-designed and properly tested [22].

Differences in educational qualifications persisted after weighting and were probably due to differences in question format between the two surveys. Including all participants, proportions with various levels of qualifications was very similar between the studies, apart from those without qualifications. There was high item non-response to the qualification question among Boost participants (9%), which probably contributes to the observed difference. In the Core survey, the interviewer provided a show card with a detailed list of qualifications. The interviewer was able to assist and to probe to ensure all qualifications were mentioned. The answer category 'no qualifications' was listed last in the Boost questionnaire: if participants without qualifications only glanced at the question, they may have deemed it irrelevant: the combined proportion of Boost participants with no qualifications or no answer was similar to the Core figure.

Questions are missed for different reasons, including being too sensitive, too cognitively demanding, or skipped in error [16]. Missing responses to smoking and alcohol questions were probably due to participants' failure to follow question routing rather than a reluctance to answer. There were very few refusals in the face-to-face interview, suggesting participants did not find these questions to be particularly sensitive. The high missing item rate for questions on household income in each year's (core) HSE confirms that participants feel able to refuse to answer questions they feel are sensitive. The shorter length of the Core booklet and the presence of an interviewer may have contributed to fewer missing items in the Core GHQ12 and social support questions compared with the Boost.

### Strengths and limitations

The main strengths of this study were the identical sampling methods and contemporaneous conduct of the two surveys in the same area. The principal limitations were the lack of information on non-responding households and the need to use mid-year estimated populations for some of the regional and PCT-level socio-demographic data for comparisons, as the Census was five years before the survey.

Although interviewers were generally assigned to only one of these two surveys, they were all employed by the same organisation and had identical general training, with differences only in the project-specific training for the specific survey they were undertaking. The main difference was that the interviewers working on the Core HSE were generally more experienced than those working on the Boost, where less interviewer interaction was required. This could have had an effect on response rates, although it is difficult to test this here. Such differences occurred because this project was a 'natural experiment' [23] rather than a planned trial.

The Boost method may be less suited to urban areas like London, where two contacts are required to obtain a productive interview (delivering and collecting the questionnaire), given that household response rates generally tend to be lower in London and other metropolitan areas than elsewhere in England. The HSE interview can be completed on first contact with all eligible adults if they are present. The high proportion of residents from minority ethnic groups may mean that English language proficiency is a more common barrier in London, since translated questionnaires were not available. The proportion of individuals in London whose proficiency in reading and writing English is insufficient to answer a self-completion questionnaire may be higher than the proportion who are unable to understand and answer spoken questions in English. There is also evidence from a number of countries in Europe for lower survey response rates in other capital cities (J Mindell, personal communication).

A number of options exist for collecting local level data. We considered and rejected a postal survey, with respondents completing something sent to them in the post, because of poor response rates, particularly in metropolitan areas, and because we wished to make the data as comparable as possible with the core survey, including a household interview and measurement of height and weight, where possible. The London Boost survey was therefore designed to have interviewers visiting the selected addresses, doing a short household interview, and leaving the self-completion questionnaires to be completed by respondents. This is a very unusual design, that not many people will follow; while it has cost savings, these are considerably less than would be obtained from a straightforward self-completion without any interviewer involvement.

## Conclusion

In summary, the individual response rates were lower in the Boost sample although those who did respond in both survey modes were very similar in terms of participant characteristics and item non-response. In an area where literacy levels in the survey language are generally adequate, using the Boost method can provide similar response rates at lower costs than face-to-face interviews, enabling larger sample sizes for the same costs. However, this may be less useful in metropolitan areas that generally have the lower survey response rates associated with a younger demographic, higher migrant populations, and therefore more non-native speakers, and greater deprivation, associated with lower literacy levels.

## Pre-publication history

The pre-publication history for this paper can be accessed here:

http://www.biomedcentral.com/1471-2288/10/83/prepub
